# Supplementary material for: Causal Relationship between Aging and Anorexia Nervosa: A White-Matter-Microstructure-Mediated Mendelian Randomization Analysis
Source: Biomedicines. 2024 Aug 16;12(8):1874. doi: 10.3390/biomedicines12081874 (PMC11351342; doi:10.3390/biomedicines12081874)
Supplement: Supplementary file 1 [file biomedicines-12-01874-s001.zip › Supplementary Figure/Figure S4/Singe SNP plot of MR analysis for TL to ubm-b-1741.pdf]

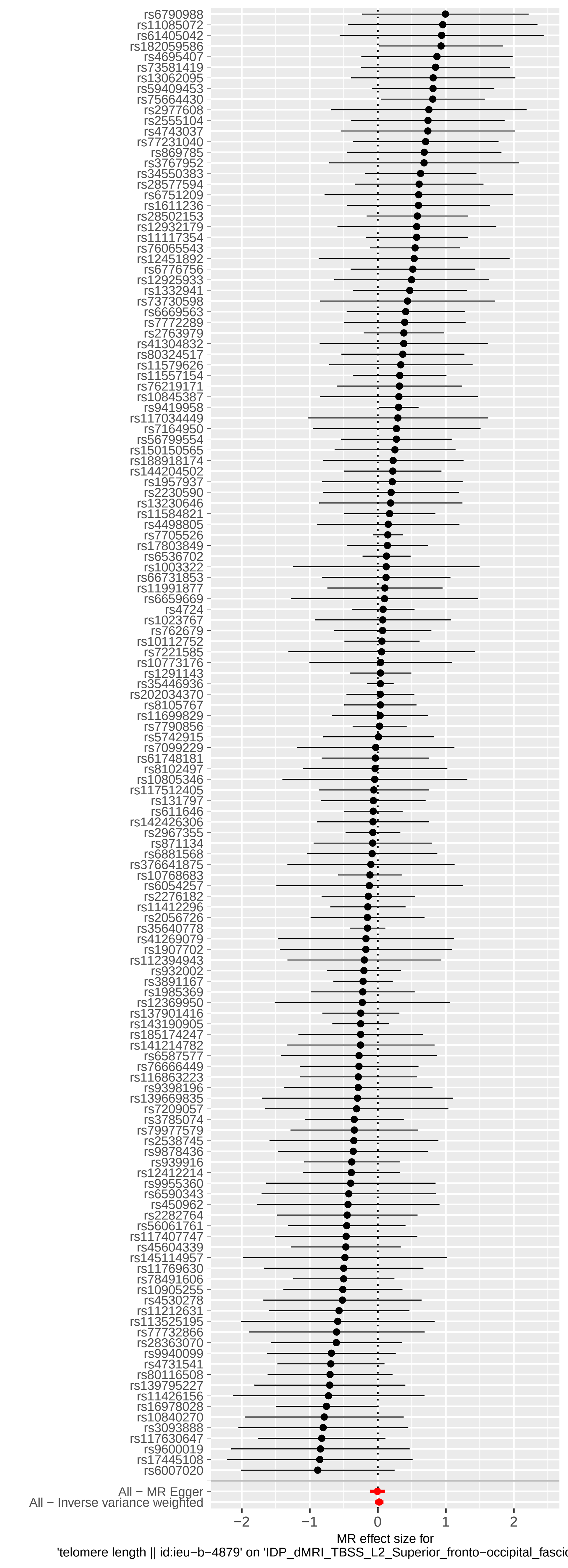
rs6790988
rs11085072
rs61405042
rs182059586
rs4695407
rs73581419
rs13062095
rs59409453
rs75664430
rs2977608
rs2555104
rs4743037
rs77231040
rs869785
rs3767952
rs34550383
rs28577594
rs6751209
rs1611236
rs28502153
rs12932179
rs11117354
rs76065543
rs12451892
rs6776756
rs12925933
rs1332941
rs73730598
rs6669563
rs7772289
rs2763979
rs41304832
rs80324517
rs11579626
rs11557154
rs76219171
rs10845387
rs9419958
rs117034449
rs7164950
rs56799554
rs150150565
rs188918174
rs144204502
rs1957937
rs2230590
rs13230646
rs11584821
rs4498805
rs7705526
rs17803849
rs6536702
rs1003322
rs66731853
rs11991877
rs6659669
rs4724
rs1023767
rs762679
rs10112752
rs7221585
rs10773176
rs1291143
rs35446936
rs202034370
rs8105767
rs11699829
rs7790856
rs5742915
rs7099229
rs61748181
rs8102497
rs10805346
rs117512405
rs131797
rs611646
rs142426306
rs2967355
rs871134
rs6881568
rs376641875
rs10768683
rs6054257
rs2276182
rs11412296
rs2056726
rs35640778
rs41269079
rs1907702
rs112394943
rs932002
rs3891167
rs1985369
rs12369950
rs137901416
rs143190905
rs185174247
rs141214782
rs6587577
rs76666449
rs116863223
rs9398196
rs139669835
rs7209057
rs3785074
rs79977579
rs2538745
rs9878436
rs939916
rs12412214
rs9955360
rs6590343
rs450962
rs2282764
rs56061761
rs117407747
rs45604339
rs145114957
rs11769630
rs78491606
rs10905255
rs4530278
rs11212631
rs113525195
rs77732866
rs28363070
rs9940099
rs4731541
rs80116508
rs139795227
rs11426156
rs16978028
rs10840270
rs3093888
rs117630647
rs9600019
rs17445108
rs6007020
All − MR Egger
All − Inverse variance weighted
−2
−1
0
1
2
MR effect size for
'telomere length || id:ieu−b−4879' on 'IDP_dMRI_TBSS_L2_Superior_fronto−occipital_fascic
